# Vaccination against Paediatric Respiratory Pathogens

**DOI:** 10.3390/vaccines7040168

**Published:** 2019-11-01

**Authors:** Sonia Bianchini, Alberto Argentiero, Barbara Camilloni, Ettore Silvestri, Anna Alunno, Susanna Esposito

**Affiliations:** 1 Pediatric Clinic, Department of Surgical and Biomedical Sciences, Università degli Studi di Perugia, 06123 Perugia, Italy; Bianchini.sonia@fastwebnet.it (S.B.); aargentiero85@gmail.com (A.A.); ettoresilvestri1981@gmail.com (E.S.); 2 Microbiology Unit, Department of Medicine, Università degli Studi di Perugia, 06123 Perugia, Italy; barbara.camilloni@unipg.it (B.C.); anna.alunno@unipg.it (A.A.); 3 Pietro Barilla Children’s Hospital, Department of Medicine and Surgery, University of Parma, 43126 Parma, Italy

**Keywords:** respiratory pathogen, vaccine, children, complication, outbreak, infection

## Abstract

Acute respiratory infections (ARIs) are extremely common in children, especially those under 5 years old. They can lead to complications, super-infection, respiratory failure, and even compromised respiratory function in adulthood. For some of the responsible pathogens, vaccines are available. This review reports current issues about vaccines against the main respiratory pathogens to highlight the available strategies to reduce the burden of paediatric respiratory disease. The optimal use of influenza, pneumococcal, pertussis and measles vaccines is required in order to reduce ARI burden. Vaccination coverage rates must be improved to achieve the full benefits of these vaccines. Recently, advances in the knowledge of respiratory syncytial virus structural biology and immunology as well as the development of new techniques to generate vaccine candidates have increased the number of promising vaccines even against this harmful pathogen.

## 1. Introduction

Acute respiratory infections (ARIs) remain one of the most common major public health threats, accounting for millions of episodes of severe acute lower respiratory infections that result in hospital admissions of otherwise healthy infants and young children worldwide [1,2,3,4,5,6,7]. One-third of the annual deaths occurring in the world are thought to be due to infectious diseases, and respiratory tract infections are responsible for 4 million deaths worldwide each year [8]. According to estimates made by the World Health Organization (WHO), pneumonia kills more children worldwide than any other disease, even more than acquired immune deficiency syndrome (AIDS), malaria and measles combined [9,10,11].

In healthy children, nasopharyngeal colonization with respiratory bacteria is a prerequisite for the development of respiratory or invasive (i.e., sepsis, meningitis) diseases [12]. Asymptomatic transient nasopharyngeal colonization with bacteria, such as *Streptococcus pneumoniae*, *Haemophilus influenzae* and *Staphylococcus aureus*, is common and decreases with age and the maturation of the immune system. Geographic region, ethnicity, season, day-care attendance, environmental factors and previous vaccinations are important determinants of bacterial colonization [13,14].

Respiratory viruses including influenza viruses, respiratory syncytial virus (RSV), human rhinoviruses (HRV), human metapneumovirus (HMPV), parainfluenza viruses, adenovirus (ADV) and human bocavirus (BoV) are responsible for approximately 35–87% of ARIs in children [15,16,17]. Viral co-infections occur in 4–33% of children hospitalized with ARIs [18,19]. Bacterial infections caused by *S. pneumoniae* and *H. influenzae* may commonly be observed in the later stages of respiratory diseases [20]. The incidence of respiratory viral/bacterial co-infection in young children ranges from 1% to 44% [21], and studies on influenza pandemics over the last 100 years have strengthened the association of bacterial super-infections and influenza infections [2,22]. In addition, pertussis and measles still represent serious medical issues with lower respiratory tract involvement in several countries.

There has recently been an increase in the number of available vaccines against respiratory pathogens recommended for children and adolescents by the health authorities, and many studies have been performed to evaluate their efficacy, safety and tolerability. The aim of this review is to report current issues about vaccines against some respiratory pathogens to highlight the available strategies to reduce the burden of paediatric respiratory disease.

## 2. Respiratory Pathogens and Available Vaccines

### 2.1. Influenza Vaccination

Influenza is a common disease that causes annual epidemics, leading to medical, social and economic problems [23,24]. Together with the elderly population, children under 5 years of age, even in the absence of underlying chronic diseases, have the highest risk of severe disease leading to hospitalization and, although rarely, to death [25]. Influenza is estimated to be the cause of approximately 374,000 hospitalizations of children <1 year of age and 870,000 hospitalizations of children <5 years of age annually [26,27,28,29,30,31,32]. Moreover, children are the most important cause of the spread of the infection in communities because they shed the virus in greater amounts and for longer periods of time than adults [33].

Many studies have demonstrated the risk of superinfection by *S. pneumoniae* and *S. aureus* during influenza, with a significant increase in the number of medical visits, drug prescriptions, and hospital admissions for respiratory disease [34,35,36]. Influenza-related complications seem to be more common in children with underlying chronic severe diseases, which is why health authorities worldwide have long recommended that these children receive the influenza vaccine every year. However, recently collected data clearly demonstrate that otherwise healthy children can also suffer from severe influenza and that the annual number of deaths is not different from that in children with chronic severe diseases [34,35].

Every year during the influenza season, together with the two subtypes of influenza A virus (A/H1N1 and A/H3N2), two lineages of influenza B viruses (B/Victoria/2/87-like and B/Yamagata/16/88-like) simultaneously circulate; in some years, the influenza B viruses are responsible for the major disease burden [37,38,39]. Unlike influenza A viruses, humans are the sole host with epidemiological relevance for influenza B viruses. Influenza B viruses evolve mainly through genetic reassortment between strains of different lineages. This allows for the escape from host immunity and the preservation of the ability to cause disease. Regardless of the lineage, influenza B infection can cause severe disease and death [39]. During influenza season, influenza C virus can circulate infecting humans, dogs, and pigs, sometimes causing severe illness and local epidemics; however, influenza C virus is less common than the other types of influenza viruses and usually it only causes mild disease in children.

In the past, recommended vaccines included only one influenza B lineage, chosen by the World Health Organization (WHO) based on surveillance data regarding the lineage that had been observed to dominate in the previous year; however, currently, quadrivalent vaccines containing both influenza B lineages represent the best influenza prevention strategy [40,41,42]. Studies have shown that the administration of quadrivalent inactivated (IIV) and live attenuated (LAIV) influenza vaccines to healthy children is effective at reducing the total burden of influenza, including preventing severe cases and saving costs due to productivity losses in parents and school absenteeism, with an acceptable level of safety and tolerability [43,44,45].

The USA Advisory Committee on Immunization Practices (ACIP) recommends influenza vaccination for the entire paediatric population, regardless of age and health conditions, starting from a minimum age of 6 months for IIV and a minimum age of 2 years for LAIV vaccines [46,47]. The ACIP highlights the importance of administering 1 dose of any influenza vaccine annually to prevent influenza disease and complications, with 2 doses separated by at least 4 weeks for children 6 months–8 years who did not receive at least 2 doses of influenza vaccine before 1 July 2018 [46]. Within Europe, there are huge variations in influenza vaccine recommendations; for example, in Italy, as in some other countries, the influenza vaccine is recommended only for at-risk people and not healthy children [48].

The need to protect neonates and infants in the first 6 months of life from influenza is so important that many health authorities have recommended influenza vaccinations of pregnant women [49,50,51]. Studies have shown that vaccination during pregnancy is effective in reducing influenza cases for at least one influenza season [52,53].

Experts agree that the available influenza vaccines can significantly reduce the risk of contracting influenza in healthy or immunocompromised subjects of any age [54]. Preventing influenza infection through vaccination may decrease the subsequent burden of infection by some of the bacterial pathogens, reduce hospitalizations, and reduce antibiotic prescriptions for influenza complications in children and adolescents [55,56]. Moreover, a reduction in influenza cases can have an impact on the abuse of antibiotics that are irrationally prescribed to a large number of paediatric patients with uncomplicated influenza, thus limiting the increase in antimicrobial resistance [57,58].

### 2.2. Pneumococcal Vaccination

*S. pneumoniae* is a Gram-positive coccus that can survive under both aerobic and anaerobic conditions [59]. It causes mucosal and invasive infections in children and adults, most commonly acute otitis media (AOM), community-acquired pneumonia (CAP), and invasive pneumococcal diseases (IPD), such as bacteraemia and meningitis [60,61,62,63,64].

In the late 1970s, a 14-valent Pneumococcal Polysaccharide Vaccine (PPV) was registered in the United States, containing purified polysaccharides of 14 pneumococcal serotypes (1, 2, 3, 4, 6A, 7F, 8, 9N, 12F, 14, 18C, 19F, 23F, and 25F) [65,66]. In 1983, this vaccine was replaced by a 23-valent PPV (PPV23) [66]. However, the polysaccharides pneumococcal vaccines were not effective in younger children and were not able to induce herd immunity.

The first polysaccharide capsular antigen conjugated to a protein (PCV) was PCV7 (4, 6B, 9V, 14, 18C, 19F, 23F), which was introduced in the United States in 2000 for use in infants. Its introduction in Europe followed in 2001. With the development of PCVs, a new era in the prevention of infections due to *S. pneumoniae* has begun [67]. However, despite being able to significantly reduce pneumococcal disease incidence, the first PCV, the heptavalent preparation (PCV7), quickly exhibited limitations. It was protected against 7 pneumococcal serotypes, considerably fewer than the total number of infective pneumococcal serotypes that circulate [67]. Moreover, the composition of PCV7 was based on serotypes that most commonly caused invasive pneumococcal disease (IPD) in the USA [67]. However, other serotypes isolated more frequently in other countries meant PCV7 could be less effective than expected in some geographic areas. To address these limitations, a few vaccines effective against an increased number of pneumococcal serotypes were developed. Among them, those with 10 and 13 serotypes were licensed for use in humans. Both contained, together with the serotypes already present in PCV7, serotypes 1, 5, and 7F [68,69]; in PCV13 serotypes 3, 6A and 19A were added [68,69]. A great number of studies have shown that both vaccines are immunogenic, safe, well tolerated and very effective because their contribution reduces the risk of pneumococcal diseases in vaccinated children and, increases herd immunity, for the unimmunized [68,69].

Practically, for the prevention of pneumococcal infections in the paediatric population it is generally inferred that PCV10 and PCV13 are similarly effective and that only the cost of vaccine procurement can influence the choice to use one over the other [70].

### 2.3. Pertussis Vaccination

*Bordetella pertussis* is a Gram-negative coccobacillus that causes whooping cough and persistent cough, especially in neonates, school-aged children and adolescents [71,72].

The classic manifestation of the disease can be divided into three phases: (1) nonspecific symptoms, such as coryza, fever, and occasional cough; (2) constant and uncontrollable cough after two weeks, followed by forced inspiration producing a whooping sound; and (3) the convalescence phase, in which symptoms decrease progressively, and complications can appear. Complications, such as pneumonia, are frequent and are responsible for over 90% of the deaths attributable to the disease in children younger than one year of age [73,74].

There has been an increase in the incidence of pertussis in the European Union from 2011 onwards [75]. The resurgence of pertussis observed in recent years seems to be a complex but real phenomenon due to a number of reasons, including the use of acellular pertussis (aP) vaccines in many locales. Lack of mucosal immune responses after aP vaccine administration favour infection, persistent colonization and transmission of the pathogen. Moreover, earlier waning of protective immunity and the circulation of *B. pertussis* variants depleted of vaccine-included antigens further favor the increase in pertussis disease [74]. Studies in immunized children have reported that antibody responses and protective immunity wane 3–5 years after immunization with acellular pertussis (aP) vaccines, which may reflect poor induction of memory T and/or B cells [76,77,78].

The majority of health authorities recommend the administration of 2–3 aP vaccine doses in the first year of life with the administration of booster doses at pre-school age, during adolescence and then every 10 years during adulthood [79,80]. Recently, in several countries vaccination against pertussis in the 2nd and 3rd vaccination during pregnancy is recommended in order to prevent pertussis in the first 6 months of life [81,82]. Studies showed > 90% effectiveness of pertussis vaccination of mothers against pertussis in the first 6 months of life. Vaccine administration in pregnancy is safe for both mother and foetus [81,82].

In conclusion, prevention of pertussis with currently available vaccines reaching high vaccination coverage rates remains a priority, including the vaccination of pregnant women [83]. Several different aP vaccines are available, but it has yet to be determined which of them confers the highest and the most-prolonged protection. Further studies are needed to evaluate the importance of individual antigens included in aP vaccines in conferring protection against disease, colonization, and transmission. However, present knowledge seems to indicate that pertussis toxin, particularly if genetically detoxified, represents the main antigen that ensures protection from disease even if not from infection. The optimal pertussis vaccine would be one that induces both mucosal and systemic responses similar to those occurring under natural infection, leading to long-term protection against both disease and infection. Such a vaccine might increase public confidence and result in better vaccine uptake.

### 2.4. Measles Vaccination

Measles is an acute viral illness caused by a single-stranded RNA enveloped virus in the family *Paramyxoviridae*, genus Morbillivirus [84,85]. It is characterized by a prodrome of fever and malaise, cough, coryza, and conjunctivitis, followed by a maculopapular rash. Measles can lead to severe complications, which can deeply impact children, and pneumonia represents one of the most common [84,85]. It is still responsible for more than 100,000 deaths every year [84,85].

Although the measles vaccine was introduced in national vaccination schedules in the majority of the countries 20 years ago, several outbreaks have occurred because of insufficient vaccination coverage in the European Region [86,87]. Every month, despite the availability of the vaccine, many measles cases continue to be reported worldwide, with episodic clusters. In the USA, 880 cases were reported from the beginning of 2019 through May [86]. In Europe, twenty-seven countries reported measles data for March 2019, with 1,548 cases reported by 22 countries [87]. These data highlight the importance of maintaining high vaccination coverage with two doses of measles vaccine administered in the first years of life, the necessity of identifying susceptible individuals of any age and considering undertaking catch-up immunization or supplementary immunization activities to close immunity gaps.

### 2.5. Respiratory Syncytial Virus (RSV)

RSV is a single-stranded RNA enveloped virus belonging to the recently named *Pneumoviridae* family, Orthopneumovirus genus, which causes lower respiratory tract illness [88,89]. Infection does not confer immunity to upper respiratory tract reinfection. The peak of severe disease is among infants in the first 3 months of life [90]. Prematurity, low birth weight, male sex, broncho-pulmonary dysplasia, congenital heart disease, immunodeficiency, cerebral palsy, and Down’s syndrome are risk factors for severe RSV bronchiolitis, but 50–80% of emergency admissions occur in otherwise healthy infants born at term [90]. Worldwide, RSV disease in children under the age of 5 years accounts for an estimated 33.8 million lower respiratory tract infections, 3.4 million hospitalizations, and up to 200,000 deaths annually [90].

Despite a consensus on the need for an RSV vaccine, there is no licensed product available yet, mainly due to the early age of infection, the capacity of RSV to evade innate immunity, and the failure of RSV-induced adaptive immunity to prevent re-infection [91]. Several clinical trials are now ongoing to assess the safety and effectiveness of different RSV vaccine candidates [92,93]. Owing to the substantial burden of RSV disease worldwide, RSV vaccine continues to be a necessity for most infants, children and also the elderly. The ideal vaccine should produce long-lasting immunity characterized by a robust Th1-mediated response and high titers of neutralizing antibodies; furthermore, it should protect against both RSV-A and RSV-B, in the presence of maternal antibodies as well, and avoid vaccine-enhanced disease.

Following the first clinical vaccine trial in the 1960s, significant progress has been made. Improved understanding of RSV immunology and structural biology as well as recent advances in vaccine technology are the bases of some of the successes. There are currently a large number of candidates in the pre-clinical phase or undergoing clinical trials, and we are waiting for the information to become available from these studies. Among the candidates in advanced clinical trials, nanoparticle and subunit vaccines are the most promising for pregnant women and the elderly, whereas live-attenuated, vector-based or subunit vaccines are being considered the paediatric population. Ongoing studies could identify effective candidates. An active instrument against infection is needed since RSV infection can cause serious complications in infants, young children and the elderly.

## 3. Conclusions

ARIs are extremely common in children, especially those under 5 years old. They can lead to complications, super-infection, respiratory failure, and even compromised respiratory function in adulthood. For some of the responsible pathogens, vaccines are available. This review focuses on the most recent data about vaccines against respiratory pathogens. The use of influenza, pneumococcal, pertussis and measles vaccines is essential to reduce ARIs burden. Vaccination coverage rates must be improved to achieve the full benefits of these vaccines. Recently, advances in the knowledge of RSV virus biology and immunology as well as the development of new techniques to generate vaccine candidates are finally increasing the number of promising vaccines against even this harmful pathogen.
